# Green Synthesis of Graphene Quantum Dots (GQDs) and Carbon Dots (CDs) Mediated with Erythrina caffra for Potential Antiviral Properties Against SARS-CoV-2

**DOI:** 10.3390/ma19091841

**Published:** 2026-04-30

**Authors:** Refilwe Matshitse, Boetie M. Buta, Nothando S. Mabasa, Bongeka S. Nkosi, Lebogang A. Ramarope, Nhluvuko Vuma, Nomusa Sikhakhane, Tebogo Matlala, Charity E. Maepa, Sifiso A. Nsibande, Daniel Makanyane, Xavier Siwe Noundou

**Affiliations:** 1 Department of Pharmaceutical Sciences, School of Pharmacy, Sefako Makgatho Health Sciences University, Ga-Rankuwa, Pretoria 0204, South Africa; boetiemlungisi@gmail.com (B.M.B.); 201805593@swave.smu.ac.za (N.S.M.); nkosibongekasiphe@gmail.com (B.S.N.); leboramarope@gmail.com (L.A.R.); vumanhluvuko19@gmail.com (N.V.); nomusa.sikhakhane@smu.ac.za (N.S.); 201608154@swave.smu.ac.za (T.M.); 2 Laboratory for Microscopy and Microanalysis, Faculty of Natural and Agricultural Sciences, University of Pretoria, Pretoria 0028, South Africa; charity.maepa@up.ac.za; 3 Chemistry Department, Faculty of Natural and Agricultural Sciences, University of Pretoria, Pretoria 0028, South Africa; sifiso.nsibande@up.ac.za; 4 Applied Radiation Department, South African Nuclear Energy Corporation Ltd., Brits 0240, South Africa; makanyanedaniella@gmail.com

**Keywords:** carbon dots, quantum dots, *Erythrina caffra*, plants extracts, SARS-CoV-2, antiviral activity and toxicity

## Abstract

This paper presents work on the green synthesis of the graphene quantum dots (GQDs) and carbon dots (CDs) from leaves of *Erythrina caffra* (*E. caffra*) using a simple technique to facilitate the carbonization process, from methanol and water extracts of *E. caffra* leaf, and their evaluation as potential antiviral agents against SARS-CoV-2. Phytochemical profiling of *E. caffra* leaf extracts exhibited the presence of phenols, alkaloids, steroids/terpenoids, tannins, and flavonoids. FTIR analysis confirmed the incorporation of oxygenated functional groups inherited from the phytochemicals. UV-Vis indicated the presence of secondary metabolites in both extracts and CDs. X-ray diffraction spectra confirmed the amorphous and crystalline nature of synthesized CDs (2.51 nm) from water extracts and GQDs (0.08 nm) from methanol extracts. The CDs and GQDs exhibited respective sizes of 5.5 and 4.0 nm, with a dot-like morphology, and respective zeta potential of +200.0 and −12.6 mV. The results revealed that all extracts and carbon dot formulations exhibited high cell viability (>90%), indicating excellent biocompatibility and minimal cytotoxicity at the tested concentration of 100 mg/mL per sample. The SARS-CoV-2 experiments demonstrated that extracts (MeOH, H_2_O) and nanomaterials (CDs-H_2_O, GQDs-MeOH) exhibited a virus suppression efficacy of 87.86 ± 4.75%, 87.95 ± 0.77%, 87.95 ± 3.08%, and 94.84 ± 0.94%, respectively. All examined samples demonstrated viral inhibition over 88%. Both extracts and their respective nanomaterials showed that a minimum of 5 μg was required to achieve 50% antioxidant species per sample. The study highlights *E. caffra* as a sustainable precursor for eco-friendly carbon dot synthesis as potential antiviral and antioxidant candidates.

## 1. Introduction

Severe Acute Respiratory Syndrome Coronavirus 2 (SARS-CoV-2) continues to cause significant morbidity, mortality, and socioeconomic disruption, thus representing a major global health concern [1,2]. The long-term efficacy of current Coronavirus Disease 2019 (COVID-19) treatment options remains limited by the emergence of resistant viral variants and adverse drug reactions, despite the availability of various antiviral agents and vaccines [3,4,5]. The SARS-CoV-2 virion is composed of four main structural proteins: Spike (S), membrane (M), envelope (E), and nucleocapsid (N) proteins [3,4,5,6]. Each of the proteins plays a significant role in viral replication [6]. The M protein is regarded as the organizer of the coronavirus assembly [7]. The key role of this M protein is to coordinate the assembly of the virus and determine the overall shape of the viral envelope by interacting with other structural proteins, such as S, E, and N protease [3].

The viral assembly and budding are mediated by the E protein [4,8], while the N protein is a phosphoprotein that protects the viral genome and is involved in viral RNA transcription, replication, and packaging the genome into new virions [5]. The S protein is a heavily glycosylated homotrimer that is conformationally flexible and plays the role of receptor-binding domain when infecting the host [5], via the S1 subunit by attaching to the host cells, which is an integral type I transmembrane protein with monocarboxypeptidase activity (ACE2) receptor, and the S2 subunit mediates fusion of the viral and cellular membrane, further enabling viral entry. Consequently, the search for alternative therapeutic strategies has intensified, with nanotechnology-based approaches showing considerable promise [9,10].

Graphene quantum dots (GQDs) [11] are a type of carbon dot (CD). They are distinguished by their ordered, graphene-like atomic structure, number of layers, and planar morphology, leading to quantum confinement effects stemming directly from their graphene lattice and their attractive properties for various applications, including high water solubility, low toxicity, photoluminescent properties, and ease of surface modification [12,13,14,15,16,17]. Carbon nanodots (CDs) are amorphous with diverse morphologies, possessing a carbon backbone with various functional groups attached to sp^2^/sp^3^ hybridized carbon atoms. The fluorescence can be attributed to either quantum confinement effects or surface defects [12,18,19]. Graphene quantum dots (GQDs), on the other hand, consist of islands of graphene-like structure, discoidal, or sheet-like morphology, and are composed of aromatic carbon, and their fluorescence is attributed to the lateral confinement within the graphene lattice [12,14,19,20]. High-surface-area and functional groups in carbon dots and GQDs improve reactivity, mechanical, and thermal robustness [12,19]. Their structure essentially determines their electrical, mechanical, optical, and chemical properties, opening a wide range of uses in nanotechnology, medicine, and energy [9,15,16,17].

Based on these qualities, they are good candidates for biological uses such as direct viral inhibition, biosensing, and antiviral medication administration [15]. A growing body of research has shown that GQDs and CDs made from natural plant sources can interact with viral proteins, inhibit replication, and strengthen host defenses [9,15,16,21]. Green synthesis techniques, which make use of renewable and ecologically friendly precursors, are known as sustainable methods for creating nanomaterials [15]. Plants provide excellent carbon sources, due to their profusion of bioactive substances, such as phenolics, flavonoids, and alkaloids, which can support the functionalization and biological activity of the resultant CDs [15]. As previously mentioned, carbon dots are prepared in this study by using leaves of *E. caffra*, a medicinal plant from South Africa. *E. caffra* is particularly chosen as no previous study has reported on this plant with respect to green synthesis of carbon dots. *E. caffra* is a native species in southern Africa that is known for its antibacterial and anti-inflammatory properties [22].

The aim of the study was to synthesize carbon dots (CDs) from *E. caffra* extracts using a heating method to induce the carbonization process and evaluate their antiviral properties against the SARS-CoV-2 papain-like protease (PLpro) [23].

## 2. Materials and Methods

### 2.1. Materials

Distilled water (Lasec purite select fusion, Serial No 33386), methanol (RLS Chemicals 99.5% AR, Lot No RLS-23137, and CAS No 67-56-1), ethanol (RLS Chemicals 99.9% AR, Lot No RLS EE0065/23, and CAS No 64-17-5), and quinine sulfate (Sigma Aldrich, Johannesburg, SA) (W/W) ≥90–≤ 100%, and CAS No 207671-44-1) were used. *E. caffra* leaves were sampled from the garden located at Sefako Makgatho Health Science University, PTA, SA. The BPS Bioscience kit (#79995-1), California, USA includes Recombinant Papain-like protease (2 µg, store −80 °C), PLPro Substrate (5 mM, 25 µL, −80 °C), PLPro Assay Buffer (25 mL, −20 °C), 10 mM GRL0617 (20 µL, stored at −80 °C), 0.5 M DTT (200 µL, −20 °C), black low-binding plate, and sealer. The HepG2 cells used in this study were obtained from the ATCC (American Type Culture Collection, Rockville, MD, USA). Cells were cultured in T75 cm^2^ tissue culture flasks using Dulbecco’s Modified Eagle Medium (DHEM) (ThermoFisher Scientific, Waltham, MA, USA) supplemented with 5% fetal Calf Serum (FCS) (Sigma-Aldrich, St. Louis, MO, USA) and 1% antibiotics (amphotericin/penicillin/streptomycin) (Sigma Aldrich, St. Louis, MO, USA). These cells were cultured and maintained in sterile culture flasks at 37 °C with 5% CO_2_ in a Forma Scientific water-jacketed incubator (ThermoFisher Scientific, Waltham, MA, USA), which provided a humidified atmosphere. Once the cells reached confluence, they were rinsed with PBS (ThermoFisher Scientific, Watham, MA, USA), treated with TrypLE Express solution (ThermoFisher Scientific, Watham, MA, USA), and subsequently added. A total of 0.1% trypan blue (ThermoFisher Scientific, Waltham, MA, USA) and a hemocytometer (Neubauer chamber) (ThermoFisher Scientific, Waltham, MA, USA) was used to count viable cells.

### 2.2. Equipment

The UV-visible (UV-vis) spectra were obtained from an Agilent Cary 60 (Agilent Technologies, California, USA) spectrophotometer equipped with an Xe lamp as a light source and a silicon diode detector. The UV-vis transmittance spectra were obtained in the spectral region of 200–800 nm. The spectra were obtained by dissolving 1 mg (extracts or nanomaterials) in 2 mL of dimethyl sulfoxide (DMSO) solvent. The pure DMSO was used as a reference spectrum in order to perform 100% transmittance correction. To operate the spectrophotometer and process data, the Cary WinUV software version 5 was used. The absorbance (A) was obtained from transmittance (T) using the A = log(1/T) expression.

The fluorescence spectra were obtained from a FluoroMax-4 (Horiba Scientific, New Jersey, USA) spectrofluorometer. The Xe arc lamp was used as an excitation source. The excitation wavelength was 325 nm. The detector was a Photomultiplier Tube (PMT). The fluorescence spectra were obtained in 90° geometry. The slit width was 5 nm. To operate the spectrofluorometer, the FluoroEssence V3.3 software was used.

The IR absorbance spectra were obtained from an FTIR spectrometer Agilent Carry 630 (Agilent Technologies, California, USA) equipped with a deuterated triglycine sulfate (DTGS) detector and an attenuated total reflectance (ATR) accessory. To operate the spectrophotometer and process data, Agilent MicroLab Software version 5.8 were used. The ATR crystal was diamond. Spectra were obtained in the 650–4000 cm^−1^ spectral region with 2 cm^−1^ resolution.

The size distribution, poly-dispersive index (PDI), surface charge, and zeta potentials were investigated using the Dynamic Light Scattering (DLS) technique with the particle size analyzer Nanotrac wave II (Microtrac MRB, Microtrac FLEX 11 Software, Osaka, Japan). The samples were dissolved in H_2_O with a refractive index of 1.333 at a temperature of 25 °C. 

The structure of the samples was studied using powder X-ray diffraction techniques with a diffractometer D2 phaser (Bruker, California, USA) equipped with a SSD160_2 (ID mode) detector, with the radiation source of Cu-Kα (λ = 1.5405 Å). The X-rays’ accelerating voltage was 30 kV with 10 mA current. The diffraction was analyzed within the 5–100° 2θ region. The data acquision and processing was conducted with Bruker DIFFRAC.SUITE and DIFFRAC.EVA software version 6.

Morphology and size of samples were determined with Transmission Electron Microscopy (TEM) JEOL JEM-2100F (Japan Electron Optics Laboratory Ltd., Tokyo, Japan). The accelerating electron voltage was 200 kV with a beam valve emission current of 149 µA. Particle diameters were measured from TEM microphotographs using Image J software version 1.5×. In each case, more than 50 particles per sample were measured.

The GloMax Explorer (Promega, Madison, USA) was used to measure bioluminescence and chemiluminescence in SARS-CoV-2. GloMax Explorer System Software version 4.2 to operate and analyze data, typically running on an integrated Windows-based tablet. The excitation wavelength was 365 nm. The emission was detected in the range of 415–445 nm using a PMT detector operating in the spectral range of 300–600 nm. Fluorescence for the resazurin assay was detected using an automated FLUOstar OPTIMA plate reader (BMG Technologies, Offenburg, Germany) with excitation of 544 nm and emission of 590 nm.

### 2.3. Extraction

The dry leaves of the *E. caffra* were milled to obtain 50 g of fine powder that was sequentially extracted for 24 h using 1 L each of both methanol and water to afford MeOH and H_2_O filtrates, respectively. The filtrates were then filtered using a funnel and Whatman filter paper to yield MeOH and H_2_O filtrates. The water filtrate was then frozen and freeze-dried to remove all the water and obtain the H_2_O (4 g, 7%) extract [24]. The methanol filtrate was evaporated under reduced pressure using a rotary evaporator to afford the MeOH (16 g, 32%) extract [24]. The two extracts were stored at 4 °C until further use.

### 2.4. Phytochemical Screening

The phytochemical screening of the various leaf extracts produced from *E. caffra* was conducted using conventional qualitative procedures derived from the literature [25,26,27,28,29,30]. For each test, 1 mg of dried plant extract was weighed and placed into six independent test tubes. In each test tube, 1 mL of either methanol or water solvent was added relative to the extract. The solutions were agitated thoroughly to ensure complete dissolution, and appropriate phytochemical reagents were added. The method was repeated three times for each phytochemical test.

#### 2.4.1. Alkaloids Detection

Two common tests, Mayer’s test and Wagner’s test, were employed to detect the presence of alkaloids in a crude extract.

1. Mayer’s Test:
  To perform Mayer’s test, 1 mL of Mayer’s solution (prepared with 1.36 g mercuric acid and 5 g potassium iodide in 100 mL of distilled water) was added to test tubes containing the crude extract and solvent. The absence of a creamy white precipitate indicated that no alkaloids were detected by this method [25].
2. Wagner’s Test:
  For Wagner’s test, Wagner’s solution was prepared by dissolving 2 g of iodine and 6 g of potassium iodide in 100 mL of distilled water. This solution was then added to each test tube. The appearance of a reddish-brown precipitate confirmed the presence of alkaloids using Wagner’s test [25,26].

#### 2.4.2. Detection of Tannins

The presence of tannins in plant extracts was determined using a ferric chloride test. This involved adding 1 mL of a 10% ferric chloride solution to test tubes containing the extracts. A positive result, indicating the presence of tannins, was observed as a change in coloration to either blue-black or brownish-green upon the addition of ferric chloride (FeCl_3_) [27,28].

#### 2.4.3. Detection of Phenols

To detect phenol compounds, the ferric chloride test was employed. The test involved adding a few drops of a neutral 5% ferric chloride solution to 1 mL of the crude extract in a test tube. The appearance of a dark green color served as an indicator of the presence of phenol compounds [27,28].

#### 2.4.4. Detection of Flavonoids

According to Bankole et al., a pink-red or red coloration upon addition of magnesium and hydrochloric acid to the extract indicates a positive result for flavonoids [29]. Shinoda test: Pieces of magnesium crystals were added to each test tube, followed by 1 mL of concentrated hydrochloric acid, and color change was observed.

#### 2.4.5. Detection of Steroids/Terpenoids

Salkowski’s test was performed to identify the presence of steroids or terpenoids. The procedure involved adding 1 mL of chloroform to a sample and mixing it thoroughly. Subsequently, 1 mL of concentrated sulfuric acid (H_2_SO_4_) was carefully introduced down the side of the test tube [30,31]. A reddish-brown color at the interface between the two liquid layers confirmed the presence of steroids/terpenoids [32].

### 2.5. Synthesis of Carbon Nanomaterials

Carbon nanomaterials (GQDs, CDs) were synthesized with slight modification using a reported method in the literature [33,34,35]. Briefly, 50 mg of the methanol crude plant extract was melted on a hot plate with continuous stirring at 200 °C for 30 min. This was followed by continuous addition of 1 mL of MeOH or H_2_O for 30 min to the respective plant extracts [33,34,35]. Thereafter, 30 mL of methanol or water was added to their respective vials and then transferred to centrifuge vials for 15 min at 40 rpm to remove all the plant extracts that did not form GQDs or CDs.

### 2.6. Physicochemical Characteristics

#### 2.6.1. Fluorescence Quantum Yields ($\emptyset_{f}$)

Fluorescence quantum yields for the green quantum dots were assessed in both water and methanol through comparative techniques outlined in previous studies [36,37]. Quinine sulfate, with a $\emptyset_{f}$ of 0.546, served as the standard when excited at the absorption wavelength of GQDs. The refractive indices of the solvents were accounted for using the following Equation (1):

(1) $$\emptyset_{f}=\;\emptyset_{f}^{Std}\;\times \;\frac{F\;A_{Std}\;n^{2}}{F_{Std}\;A{\;{(n}_{Std})}^{2}}$$

where n and $n_{Std}$ represent the refractive indices for GQDs and the standards, respectively. F and $F_{Std}$ denote the areas under the fluorescence curves for both the sample and standard. Meanwhile, $\emptyset_{f}$ and $\emptyset_{Std}$ refer to the fluorescence quantum yields of the sample and standard, respectively [37]. Lastly, A and $A_{Std}$ are the absorbance values for both the sample (GQDs and CDs) and standard at their respective excitation wavelengths 275 and 410 nm.

#### 2.6.2. Powder X-Ray Diffraction

The chemical and molecular structure of both the extracts and their corresponding nanomaterials was analyzed. X-ray diffraction patterns were recorded across an angular range of 5 to 90 degrees. The system employed a Cu/Ka radiation source with a wavelength of 0.154 nm, operating at 10 mA with an accelerating voltage of 30 kV. The principal diffraction peaks were integrated, and these were used to determine the crystalline index (CI%) of the samples, calculated using Equation (2):

(2) $$CI\;\left(\%\right)=\;\frac{I_{c}}{I_{c}+\;I_{a}}\;\times 100\%$$

where I_c_ (a.u.) and I_a_ (a.u.) represent the integrated intensities of crystalline and amorphous regions, respectively.

The Scherrer equation was used to calculate the crystal size, *D* (nm) at (200) plane for both CDs and GQDs using Equation (3), following previous studies [18]:

(3) $$D\;=\frac{K\lambda }{\;\beta \;cos\theta }$$

where *D* is the crystal size, K is the correction factor assigned a value of 0.9, λ is the radiation wavelength, θ is the diffraction angle in radians, and θ is the corrected angular width at the half maximum intensity in radians.

Bragg’s equation was used to calculate interlayer spacing using Equation (4) at the highest peak intensity of CDs and GQDs [38,39]:

(4) $$d=\;\frac{n\lambda }{2Sin\theta }\;$$

where n is the order of diffraction, which is a positive integer. Wavelength (λ) of the incident Cu Kα X-ray radiation of approximately 1.54184 Å, the interlayer spacing (*d* spacing), represents the distance between successive parallel planes of atoms in the crystal lattice, and θ is the angle of incidence [39]. Theoretical fit was processed with the GaussAmp function, as shown in Supplementary Data S1(a,b).

### 2.7. Pharmacological Characteristics

#### 2.7.1. Antioxidant

The DPPH (2,2-diphenyl-1-picrylhydrazyl) antioxidant assay was used to evaluate the free radical scavenging activity of samples following a previous method [40,41], quantified by measuring the decrease in absorbance [42,43,44,45]. Briefly, reagents were prepared by making a stock solution of DPPH (1 mg) in DMSO (50 mL). Sample solutions were prepared by dissolving 1 mg of the test samples in 1 mL DMSO. A mixture composed of a 1:1 ratio of DPPH and test sample solution was monitored with a UV-Vis spectrometer at 517 nm for a duration of 5 min per sample at 1 min interval [43,46].

The percentage of DPPH scavenging activity was calculated using Equation (5):

(5) $$DPPH\;Sacavenging\;activity\;(\%)=\frac{{Absorbance}_{control}-{Absorbance}_{sample}}{{Absorbance}_{control}}\times 100$$

where ${Absorbance}_{control}$ is the absorbance of the DPPH solution without the sample, and ${Absorbance}_{sample}$ is the absorbance of the DPPH solution with the test sample [46]. The percentage of DPPH radical scavenging activity is calculated using Equation (5), following previous studies [46,47]. The IC_50_ value, representing the concentration of the sample required to scavenge 50% of the DPPH radicals, can be determined from the dose–response curve [47].

#### 2.7.2. Toxicity

Human liver hepatocellular carcinoma (HepG2) cell lines were chosen to evaluate the toxicological effects of extracts and nanoparticles on cells. Cell viability was determined using the Alamar Blue (resazurin) assay with minor modifications [48]. Cells were treated with 10 µL of the respective samples and incubated for 24 h at 37 °C in a humidified atmosphere containing 5% CO_2_ in a Forma Scientific water-jacketed incubator (ThermoFisher Scientific, Waltham, MA, USA). After incubation, 11.1 µL of 2% resazurin solution was added to both treated and control wells, and the plates were further incubated for 3 h. Following incubation, fluorescence was measured using an automated FLUOstar OPTIMA plate reader (BMG Technologies, Offenburg, Germany) at an excitation of 544 nm and emission of 590 nm [48].

The percentage cell viability was calculated using the following Equation (6):

(6) $$Cell\;viability\;(\%)=\frac{{Fluorescence}_{sample}}{{Fluorescence}_{untreated\;control}}\times 100$$

#### 2.7.3. SARS-CoV-2 Papain-like Protease (PLpro) Enzyme Assay

Papain-like protease assay using fluorescence resonance energy transfer (FRET) was used to measure the ability of plant extracts and respective QDs to inhibit the SARS-CoV-2 PLpro as previously described [49,50,51,52]. Briefly, 1 mM dithiothreitol (DTT) was prepared by adding 20 µL of 0.5 M DTT stock solution to 10 mL of PLpro assay buffer. The PLpro enzyme (0.7 ng) was diluted in 1 mM DTT solution, and 30 µL was transferred to 96-well plates for test samples, positive and inhibitor controls; with the exception three wells, in which 30 µL of 1 mM assay buffer was added and referred to as the blank. A positive control (500 µM) was prepared from 10 µL of a known PLpro inhibitor (GRL0617). The FRET-based protease assay was initiated by first incubating the 96-well plate for 30 min at 37 °C after combining a buffer with PLpro (9 ng). Meanwhile, 125 μM PLPro fluorogenic substrate was prepared from a 1:40 ratio of 5 mM PLpro substrate in 1 mM PLpro buffer solution. The 96-well plate was removed from the incubator, 10 µL of the PLpro substrate was added onto the plate, and it was further returned to the incubator for an additional 60 min. Protease percentage inhibition is calculated according to Equation (7):

(7) $$Inhibition\;(\%)=(1-\frac{RFU_{sample}}{RFU_{Negative\;control}})\times 100$$

where *RFU_sample_* is the average fluorescence of inhibitor-treated wells, and *RFU_negative control_* is from enzyme-only wells without inhibitor [49]. This is determined following blank subtraction from triplicates for analysis [49].

## 3. Results and Discussion

### 3.1. Phytochemical Screening

Phytochemical screening was conducted on methanol (MeOH) and water (H_2_O) extracts derived from the *E. caffra* plant, utilizing various tests outlined in Table 1. For each test, controls were prepared using the respective extracts dissolved in their corresponding MeOH and H_2_O solvents. *E. caffra* extract of MeOH and H_2_O tested positive for alkaloids, flavonoids, tannins, steroids/terpenoids, and phenols. However, the H_2_O extract tested negative for flavonoids. Flavonoids are a diverse group of plant secondary metabolites, and they are more soluble in methanol than in water due to their chemical structure and the differing polarities of these solvents [53,54].

Flavonoids generally exhibit low solubility in water because they have a limited capacity to form hydrogen bonds with the surrounding water molecules [53]. Many flavonoids possess hydrophobic ring structures, which inherently limit their solubility in highly aqueous (water-based) solutions [54]. The hydrophobic ring structures in flavonoids limit their solubility in highly aqueous (water-based) solutions [54]. Hence, the absence of flavonoids in the H_2_O extract.

### 3.2. UV-Vis Absorbance Spectroscopy

**Figure 1:**
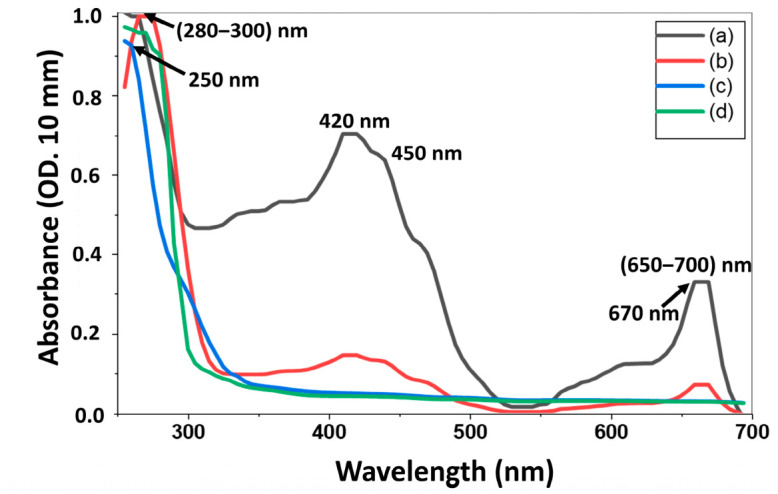
Absorption spectrum of extracts from (200–700) nm for: (a) MeOH, (b) GQDs-MeOH, (c) extract H_2_O, and (d) CDs-H_2_O.

Figure 1 shows absorption bands at 250 nm, (280–300) nm, and 670 nm for the H_2_O extract and at 250 nm, 420 nm, 450 nm, and 670 nm for the *E. caffra* MeOH extract. The 670 nm absorption bands in Figure 1a,b show the *E. caffra* methanol extract and its corresponding GQDs. Nevertheless, there was no absorption in the same area for the water extract and the corresponding CDs in Figure 1c,d. Chlorophylls, which are insoluble in water and usually absorb in the 650–700 nm range [55], are characterized by this peak. Chlorophyll, along with other phytochemicals such as tannins, flavonoids, and phenols, was found in the methanol extract, which added to the absorbance profile required for green nanomaterial fabrication. Other plant extracts, such as *Erythrina sacleuxii*, have shown distinct absorption maxima at 262 and 290 nm, which is characteristic of phenolic and flavonoid skeletons [56]. Tannins and chlorophylls have been reported to absorb around 640 and 650–700 nm [55]. A broad absorption peak at 530 nm was reported in *Erythrina crista-galli* and attributed to anthocyanins [55,57,58,59]. Clear alterations in absorption patterns are visible in the UV-Vis spectra of *E. caffra* extracts and carbon nanomaterials. Nanomaterials: these shifts correspond to the establishment of surface functional states and the change in molecular composition during carbonization. The MeOH extract in Figure 1b shows considerable absorption, a characteristic of π–π* transitions from conjugated aromatic systems, mainly in the UV area below ~300 nm. Increased absorbance at ~280–320 nm appears upon conversion to GQDs, which is compatible with additional n–π* transitions that are caused by carbonyl (C=O) or imine (C=N) groups on the surface. Strong deep-UV absorption bands (~220–260 nm) with tails into higher UV are also visible in the H_2_O extracts, as shown in Figure 1c, indicating the presence of many phenolic/flavonoid moieties. Prominent features and shoulders below 300 nm in Figure 1d with a broad absorption suggest novel surface states and functional groups from carbonization. In other studies, on plant-derived CDs, the formation of π-π* transitions (~250–300 nm) and n-π* transitions (usually ~320–350 nm) has been attributed to aromatic/sp^2^ domains and surface groups that contain either nitrogen or oxygen, respectively (e.g., in edible mushroom CDs showing peaks around 280 and 320 nm) [60]. Hence, the peak broadening observed in the UV spectra is associated with aromatic structures of CDs.

Thus, *E. caffra* in Figure 1 confirms the presence of various phytochemicals such as flavonoids, phenolics, tannins, and chlorophylls, as shown in Table 1, which would contribute to the overall absorbance profile across the UV and visible light spectrum.

### 3.3. Fluorescence

**Figure 2:**
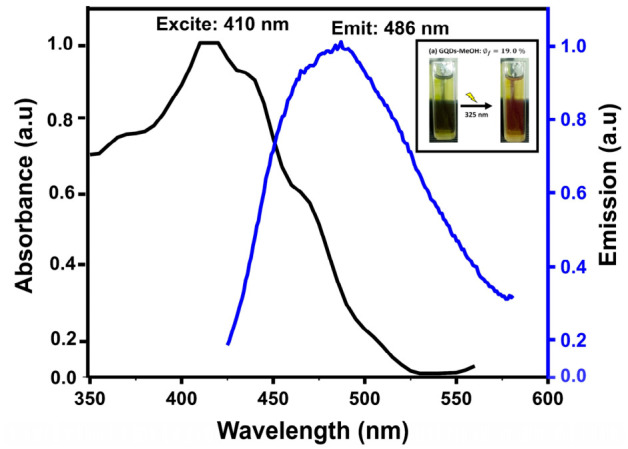

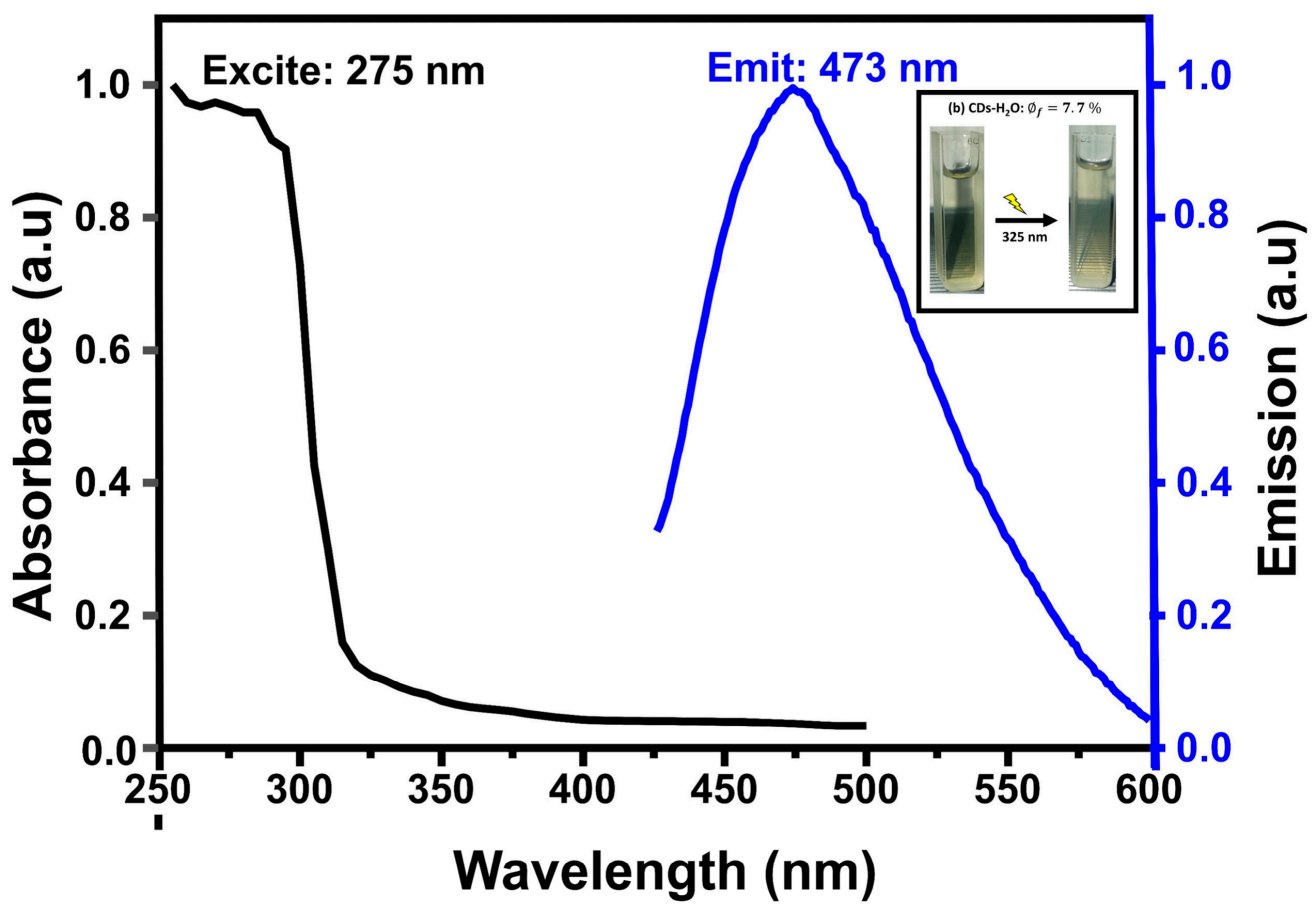
Fluorescent image of GQDs when excited at 410 and 275 nm: (**a**) GQDs-MeOH and (**b**) CDs-H_2_O.

When excited at 325 nm, both carbon nanomaterials exhibited emissions that varied in color, as illustrated in the inserted images of Figure 2. Specifically, a red emission color was observed for GQDs-methanol (GQDs-MeOH) in Figure 2a, while Carbon Dots-water (CDs-H_2_O) displayed a pale yellow emission in Figure 2b. Upon excitation of these carbon nanomaterials at their respective absorption maxima, emission was detected at 486 nm for GQDs-MeOH and 473 nm for CDs-H_2_O. The red emission color correlates with the presence of chlorophyll a pigment according to the UV-Vis absorbance spectroscopy (see Figure 1a,b). The red emission originates from chlorophyll moieties that become integrated into the carbogenic core during synthesis [61]. The calculated emission efficiencies for these nanomaterials were 19% and 7%, respectively. The red emission color correlates with the presence of the chlorophyll a pigment according to the UV-Vis absorbance spectroscopy (see Figure 1a,b). Green carbon-based quantum dots of different fluorescent quantum yields have been previously reported from various plant leaves, such as Aloe vera, pineapple, and orange [62]. However, these nanomaterials are synthesized for the first time in this study using *E. caffra* leaf extracts, and the MeOH extracts generated more photostable GQDs with high fluorescence efficiencies compared to CDs from the H_2_O extract. Fluorescence efficiencies in GQDs have been reported to be higher than those of CDs because both carbon-based nanomaterials have different structural characteristics [9,12,14,18,19]. Hence, the fluorescence quantum yields in GQDs are due to the lateral confinement within the graphene lattice, compared to surface defect quantum confinement in CDs.

### 3.4. Fourier Transform Infrared Spectroscopy (FTIR)

**Figure 3:**
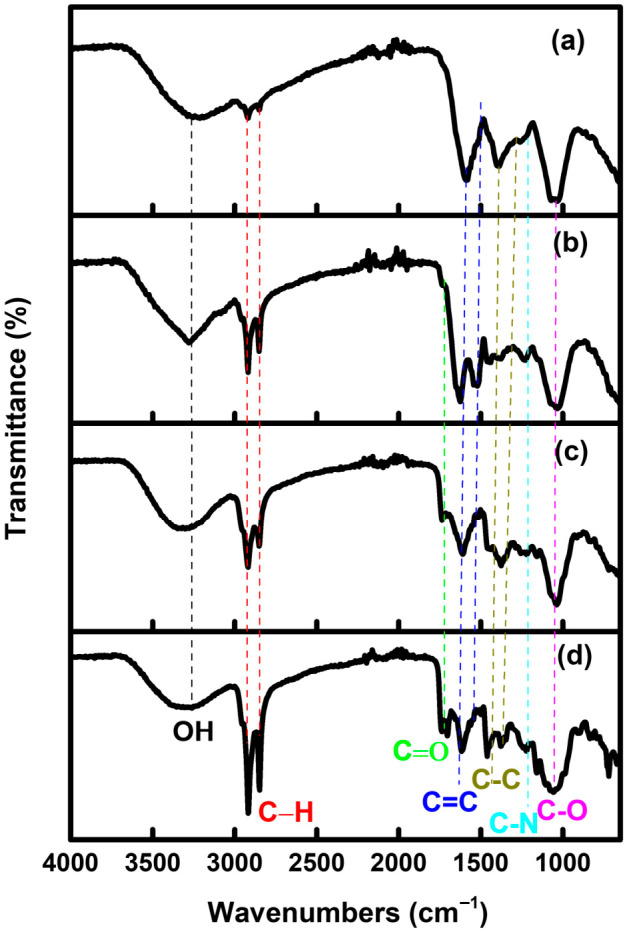
FTIR spectra of (**a**) H_2_O extract, (**b**) CDs-H_2_O, (**c**) MeOH extract, and (**d**) GQDs-MeOH.

Figure 3a–d show the absorption bands for the H_2_O extract, CDs-H_2_O, MeOH extract, and GQDs-MeOH. Functional groups of corresponding nanoparticles and extracts at FTIR frequencies (C–N) 1052.56 cm^−1^, (C–O) 1234.61 cm^−1^, (C=O) 1737.87 cm^−1^, and (C–H) 2919.68 cm^−1^ are shown in Figure 3. Peak broadening and shifting of C=C at 1594,94 cm^−1^ in H_2_O extracts compared to a narrow, split (C=C; 1631.54, 1529.26 cm^−1^), and enhanced (C–N, 1234.61 cm^−1^) CDs-H_2_O are shown in Figure 3a,b. However, Figure 3c,d show that GQDs-MeOH exhibit comparable peak increase (C=O, 1737.87 cm^−1^), shift (C=C, 1607.14 cm^−1^), split (C–C, 1453.25, 1355.66 cm^−1^), and broadening (C–O, 1052.56 cm^−1^) as the corresponding MeOH extracts; this further confirms the chemical alteration.

The absence of peak shift may result from functional group inheritance, effective surface passivation, and a steady chemical environment. This study validates that the synthesized CDs and GQDs “inherited” their surface chemistry directly from the phytochemicals found in the *E. caffra* leaves. In the process of heating, the plant metabolites (phenolics, alkaloids, and terpenoids) serve as both the carbon precursor and the agents that cap and stabilize the carbon. The plant metabolites stay on the surface of the nanoparticles as a passivation layer, and they keep the same vibrational frequencies that they had in the original extract [63,64].

The continuity of these peaks signifies that the chemical environment of the functional groups did not experience substantial structural alterations after carbonization C–H (2919.68 cm^−1^): The aliphatic chain lengths from terpenoids are predominantly unaffected by core carbonization, indicating their presence on the outside shell of the nanoparticles [65]. In the green synthesis of nanoparticles from plant extracts such as *E. caffra*, the preservation of aliphatic chain lengths from terpenoids is a critical indicator of the material’s core–shell structure [66]. During the carbonization process, the phytochemicals experience selective change, with reactive functional groups forming the core and stable aliphatic segments remaining on the surface [62]. The consistency of the carbonyl peak at C=O (1737.87 cm^−1^) verifies the presence of oxygenated moieties (such as esters or ketones), which are crucial for the elevated water solubility and biocompatibility of CDs–H_2_O [63]. The absence of a change in the C–N bond suggests that nitrogen-containing groups from the alkaloids were effectively integrated into the nanoparticle surface without substantial alteration of bond length [65]. In green synthesis, precursor molecules typically envelop the resultant carbon core via non-covalent interactions or slight structural modifications. This produces “common functional groups” between the extract and the nanoparticles, resulting in nearly identical FTIR fingerprints [58]. Comparable findings have been shown in other green synthesis investigations, wherein the peaks for hydroxyl, carbonyl, and aromatic groups in the plant matrix closely correspond to those on the surface of the resultant quantum dots [63,65]. The continuity of these peaks signifies that the chemical environment of the functional groups did not experience substantial structural alterations after carbonization.

In the context of green synthesis, the emergence, disappearance, or shifting of FTIR peaks between plant extracts and their corresponding nanoparticles (CDs–H_2_O and GQDs–MeOH) provides direct evidence of the biochemical transformation and surface functionalization occurring during synthesis. Significant shifts in the absorption bands of extracts and respective nanomaterials shown in Table 2 are a diagnostic indicator that the phytochemicals in the plant extract were actively involved in the reduction and stabilization of the nanoparticles [65].

A shift indicates a change in the chemical environment or bond energy of the functional group. In green synthesis, this typically signifies that the phytochemicals (like phenolics) are binding to the nanoparticle surface to act as capping agents, which prevents particle aggregation and ensures colloidal stability [65]. Disappearance of specific peaks from the *E. caffra* extract indicates that those functional groups were consumed or chemically modified during the reaction process [65]. For example, the loss of certain O–H or specific C–O signals may reflect the dehydration or oxidation of plant metabolites, as they provide the electrons necessary to reduce metal ions or as they undergo thermal decomposition to form the carbon core [65].

The appearance of new peaks—specifically related to C=C and C–C groups—indicates that these functional groups were formed during the synthesis and were not present in the original plant material [65]. The emergence of aromatic C=C stretching is a hallmark of successful carbonization, signifying the development of the sp^2^ hybridized carbon network that constitutes the core of CDs and GQDs [63]. Emergent peaks can also represent new surface functionalities created during the microwave-assisted or thermal process, which contribute to the nanoparticles’ unique optical and biological properties [65]. Therefore, peak shift indicates reduction and surface capping [65]. Meanwhile, peak disappearance can be associated with the consumption or decomposition of precursor metabolites [65]. Peak emergence is associated with the formation of the graphitic carbon core and new chemical bonds [63,65]. Thus, spectroscopic changes confirm that the synthesis process successfully restructured the *E. caffra* metabolites into functionalized nanomaterials that retain specific “inherited” groups while developing new structural characteristics essential for their intended applications.

The water extract in Table 2 shows that functional groups (C–N, C–H, and O–H) indicate a rich diversity of alkaloids, terpenoids, and phenolics [65,67]. These groups are essential for the biochemical properties of the extract, as they provide the necessary surface chemistry for potential interactions with biological targets or the stabilization of synthesized nanomaterials [65,68]. The peak assignment is supported by the identification of aromatic material. The C–N groups at 1392.25 cm^−1^ in the H_2_O extract originate from alkaloids that contain secondary metabolites often present in *Erythrina* species and other medicinal plant extracts used for nanoparticle stabilization [65].

The 1619 cm^−1^ peak reflects a complex mixture of aromatic metabolites (alkaloids/phenols) via C=C ring stretching [68]. These nitrogen- and oxygen-containing functional groups are essential for the biological activity of the extract and serve as the chemical interface for subsequent green synthesis applications [65].

Absorption bands in the 1200–1250 cm^−1^ range are characteristic of C–O stretching vibrations in esters, ethers, and carboxylic acids [69,70]. This aligns with phytochemical findings of phenols and tannins in the [69] *E. caffra* matrix. This wavenumber also corresponds to the in-plane bending of O–H groups [71]. Given that *Erythrina* species are rich in polyphenols, this peak reflects the high concentration of hydroxylated aromatic rings. In medicinal plant extracts, this region is frequently assigned to C–N stretching from aliphatic amines [69,70]. This supports the presence of alkaloids, which were positively identified in our phytochemical screening for both water and methanol extracts.

The persistence of these peaks indicates that the chemical environment of the functional groups did not undergo fundamental structural changes during carbonization. The C–H (2919.68 cm^−1^) is an aliphatic stretch from terpenoids that remain largely unaffected by the core carbonization, suggesting they reside on the outer shell of the nanoparticles [65]. The stability of this carbonyl peak C=O (1737.87 cm^−1^) confirms the retention of oxygenated moieties (like esters or ketones), which are essential for the high water solubility and biocompatibility of our CDs–H_2_O [63,72].

### 3.5. Powder X-Ray Diffraction (PXRD)

**Figure 4:**
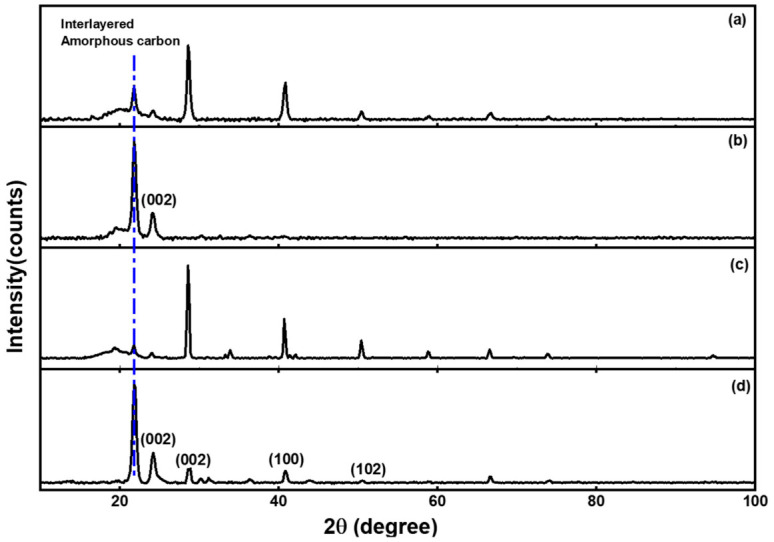
PXRD results for (**a**) H_2_O extract, (**b**) CDs-H_2_O, (**c**) MeOH extract, and (**d**) GQDs-MeOH.

Crystallographic peaks in Figure 4a–d were observed at (22, 24, 29, 41, 51)°, (22, 24)°, (22, 24, 29, 41, 51)°, and (22, 24, 29, 41, 51)° and were assigned Miller indices of (002), (002), (100), and (102) for extract H_2_O, GQDs H_2_O, extract MeOH, and GQDs MeOH. The H_2_O extract showed 100% amorphous nature in Table 3, with only 5% of the defects in the MeOH extract. The CDs and GQDs are both types of fluorescent carbon nanomaterials, but they possess distinct structural and optical characteristics. The primary differences arise from their structural makeup, which influences their size, crystallinity, interlayer spacing, and fluorescence quantum yield [12,13,14]. The CDs-H_2_O sample retained the 100% amorphous nature of its parent extract, exhibiting no crystallinity. Sharp, narrow peaks in the PXRD pattern illustrated in Figure 4a contradict the 100% amorphous crystallinity percentage presented in Table 3. This discrepancy typically originates from the characteristics of the sample. In green synthesis utilizing *E. caffra*, a composite material comprising nanoparticles embedded within a polymeric protein–phytochemical matrix is created [73]. Crystalline impurities are characterized by “sharp, narrow peaks” derived from the minerals in plant extract [74], since they constitute a minimal mass fraction relative to the bulk amorphous plant extract matrix [75]. However, the GQDs-MeOH sample achieved 100% crystallinity. This indicates that the phytoconstituents in the MeOH extract acted as superior carbon precursors, facilitating a synthesis pathway that favored the assembly of a highly ordered, crystalline nanomaterial. A more pronounced sharp peak around 22° 2θ was observed for GQDs compared to both extracts (illustrated with a blue vertical line). The increased peak intensities at 22° in both CDs H_2_O and GQDs MeOH can be associated with the presence of interlayered amorphous carbon. Consistent with its amorphous nature shown in Table 2, the CDs-H_2_O presented a broad peak from around 20°, while the GQDs-MeOH did not exhibit such broadening. The slight contraction observed in the synthesized GQDs is a common phenomenon in such nanomaterials and is frequently attributed to the introduction of oxygen-functional groups and lattice strain [76,77]. The interlayer spacing (*d*-spacing) of CDs (4.07 nm) and GQDs (4.06 nm) is higher than the typical graphitic interlayer spacing of 0.335 nm. Depending on the specific precursor and synthesis conditions, the interlayer spacing of GQDs and CDs synthesized from plant extract precursors typically ranges from 0.32 nm to 0.40 nm [14,78]. This value is typically marginally greater than the *d*-spacing of bulk graphite (0.335 nm), which is frequently attributed to the presence of oxygen-containing functional groups and a lower degree of crystallinity [12,78]. The higher values of *d* spacing in both nanoparticles result from the intercalation of oxygen-functional groups (confirmed by FTIR in Figure 3) and other structural defects between the graphitic interlayers.

Crystal sizes were calculated using Equation (3) of the diffraction peak (002) to yield 80.9 and 80.7 nm for GQDs and CDs.

### 3.6. Morphology and Size

**Figure 5:**
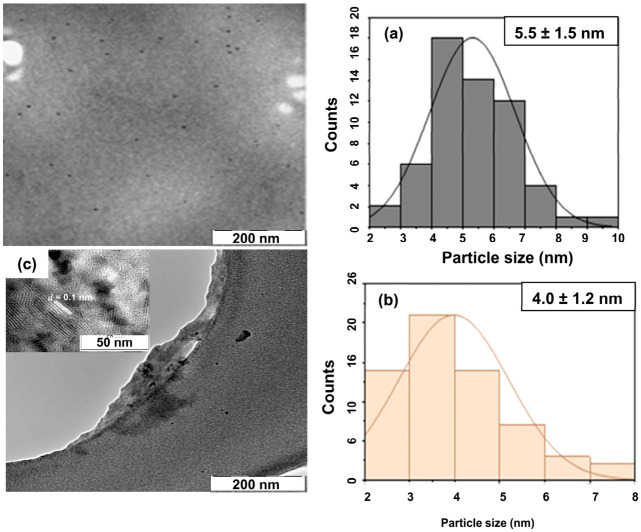
(**a**): CDs-H_2_O; (**b**) GQDs-MeOH with size distributions; and (**c**) insert of GQDs-MeOH with d spacing.

The TEM microphotographs in Figure 5a show that the *E. caffra* H_2_O-derived carbon dots are uniformly distributed, spherical, and well-dispersed without signs of aggregation. The corresponding size distribution (Figure 5a) indicates a particle diameter of 4.0 ± 1.2 nm range. Figure 5b illustrates that the MeOH extract-derived GQDs are also mostly spherical and well-dispersed, but they exhibit larger sizes of 5.5 ± 1.5 nm with a PDI of 0.2, as shown in Table 2. Lattice fringes with d spacing of 0.08 nm on an insert in Figure 5c for GQDs-MeOH are indicative of the facet of graphite [79]. Both newly synthesized nanomaterials demonstrated relatively narrow, Gaussian-like size distributions, indicating good uniformity. The Shapiro–Wilk test was conducted on a nanoparticle population that is above 50 (*n* > 50) at 95% confidence interval; see attached Supplementary Data S2(a,b). The distribution of nanoparticles is statistically normal: *p* > 0.05. These observations are consistent with the literature stating that CDs derived from biomass or plant extracts typically display dot-like morphologies, good dispersibility, and sizes ranging from a few nanometers up to several tens of nanometers, depending on the precursor and the synthesis method [15,16,33,80,81,82]. Larger sizes observed in DLS measurements for CDs and GQDs are primarily due to the inclusion of the hydrodynamic layer (hydration shell) and the high sensitivity of DLS to particle aggregation [83,84,85]. In contrast, the projected size measured by TEM represents individual nanoparticles deposited on dried grids under vacuum [86,87]. Size variations from PXRD compared to TEM are attributed to the average value of a bulk sample representing a large population of crystallites, while TEM gives a direct image of an individual particle [88,89,90].

### 3.7. Zeta Potential and Surface Charge

The zeta potential analysis in this study revealed contrasting surface charges among the *E. caffra* extracts and respective nanomaterials, highlighting the influence of solvent and carbonization on colloidal stability. Table 2 shows that the H_2_O extract exhibited a strongly negative charge of −63.6 mV, consistent with the presence of abundant anionic functional groups, whereas the methanol extract showed a positive surface charge (+49.8 mV), reflecting stabilization by cationic phytochemicals. The H_2_O-derived CDs displayed a remarkably high positive zeta potential of +200.0 mV, indicating substantial surface modification during carbonization, likely due to protonated nitrogen-containing groups, while the methanol-derived GQDs maintained a moderately positive charge.

Comparatively, these results indicate that carbonization significantly reduces particle size and alters surface charge, enhancing colloidal stability, while solvent choice during extraction influences both the initial zeta potential and particle size distribution. Overall, most samples exceeded the ±30 mV threshold for stability, demonstrating their potential for applications in biomedical fields [91,92], except for GQDs-MeOH with +12.6 mV. GQDs are generally considered to have low cytotoxicity but excellent biocompatibility [93,94]. A positive correlation between increased zeta potential and high toxicity has been reported in some CDs, but it is not a universal rule [95]. Negatively charged zeta potentials ranging from −44.1 to −9.7 mV were reported to have low toxicity [95]. And some positively charged CDs, ranging from +11.4 to +41.4 mV, reported high toxicity [95]. Negatively charged GQDs with zeta potentials of −15 ± 1.5 and −32 ± 4 mV have been reported to be very stable [96,97]. The most stable positively charged GQDs were reported at +26 ± 5 mV.

### 3.8. Quantitative Antioxidant: DPPH Scavenger Assay

**Figure 6:**
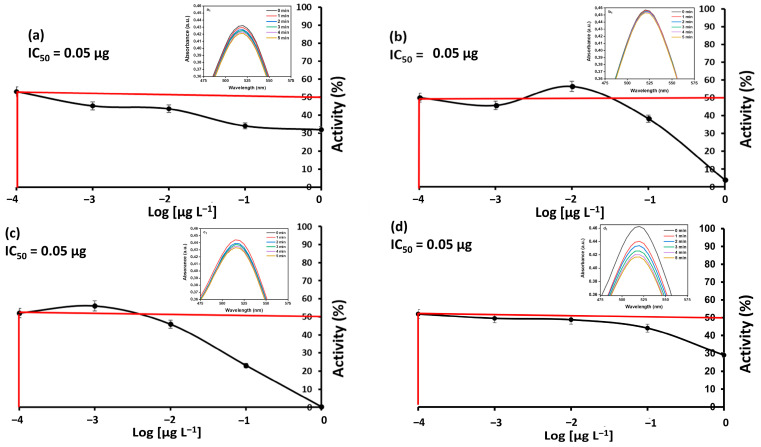
Antioxidant assay and absorbance inserts of (**a**) MeOH extract, (**b**) GQDs-MeOH, (**c**) H_2_O extract, and (**d**) GQDs-H_2_O. The red line is necessary to show the concentration that generates 50% antioxidant species.

Inserts in Figure 6(a1–d1) show that decreasing absorbance occurs as the DPPH radical reacts with antioxidants in the test sample. This decrease in DPPH over time could occur through two possible routes: hydrogen atom transfer or single electron transfer [98]. In a hydrogen transfer (dominant mechanism), the hydrogen atom from the test sample is donated to the DPPH radical, resulting in a reduced DPPH-H molecule and an antioxidant radical [99,100]. Meanwhile, in a single electron transfer, antioxidants donate a single electron to the DPPH radical to form a DPPH anion [99,100]. This anion then undergoes protonation from the solvent or another available species to form DPPH-H [43,100]. Furthermore, IC_50_ was calculated for the respective samples shown in Figure 6a–d with a change in percentage activity at concentrations (0.05, 1, 10, 100, and 1000) µg L^−1^. Both extracts and nanomaterials showed that a minimum concentration of 0.05 µg L^−1^ test samples is required to yield the respective 50% antioxidant activity.

### 3.9. Biosensing Applications: In Vitro Assays

#### 3.9.1. Toxicity: Human Hepatoma: Resazurin Assay

**Figure 7:**
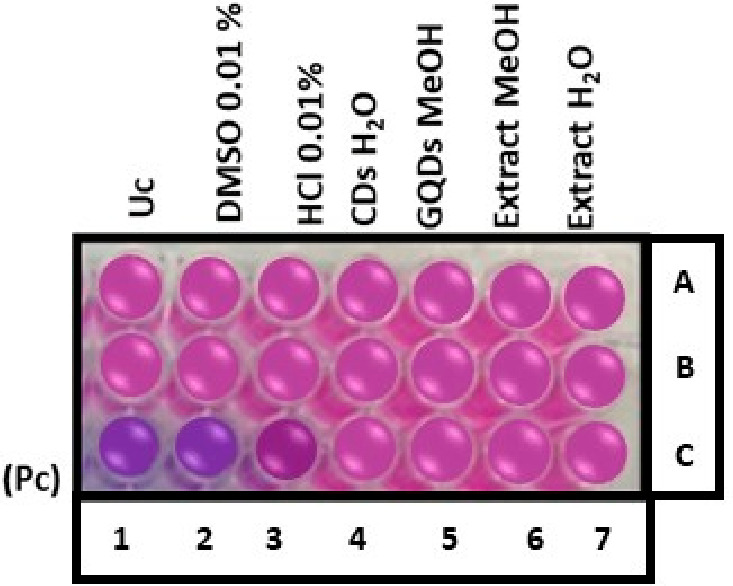
Resazurin assay on samples (extracts (A6, B6, C6, A7, B7, C7) and respective nanomaterials (A4, B4, C4, A5, B5, C5) and controls (Pc (C1–C3); Uc (A1, B1); DMSO (A1, B1); HCl (A1, B1)).

The resazurin assay, often used with HepG2 cells (Human hepatoma), is a well-established method for evaluating cell viability, metabolic activity, and cytotoxicity of living cells [101]. Resazurin is a blue non-fluorescent dye that is actively taken up by metabolically active cells and reduced to resorufin, which is pink and highly fluorescent, as shown in Figure 7. Controls used in the assay are negative control (Uc): PBS, vehicle control 1: PBS: DMSO 0.01%, vehicle control 2: PBS: HCl: MeOH 0.01%, and positive control (Pc): 1% Triton X. The highest activity was observed with a color change to blue when Pc, compared to other controls, was applied to the cell lines. The tested samples are extract H_2_O, CDs H_2_O, extract MeOH, and GQDs-MeOH. The reduction in the dye acts as a biological sensor, with the color change or fluorescence intensity indicating the viability and metabolic state of the cell [102]. The cyto-sensors use living biological cells as sensing elements to detect changes caused by external stimuli, such as toxins [103]. Thus, providing insights into the physiological effects of analytes and serving as valuable tools for drug evaluation and toxicity detection, as shown in Figure 7.

**Figure 8:**
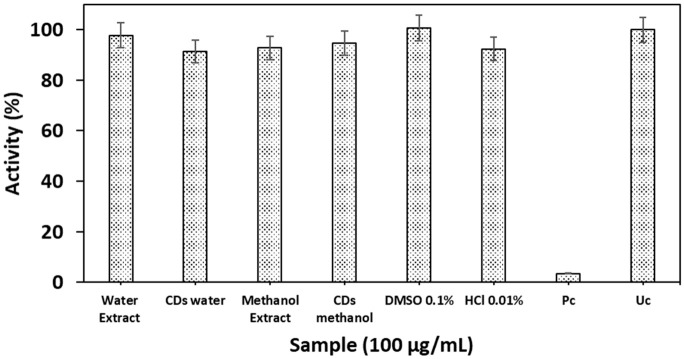
Toxicity results of *E. caffra* synthesized nanoparticles and their extracts from water and methanol.

The resazurin assay on HepG2 cell lines in Figure 8 shows minimal toxicity, with percentages that are more than 90% for all the tested samples when compared to the blank control, which showed 100%. However, MeOH extract, H_2_O extract, MeOH GQDs, and CDs-H_2_O showed respective cell viabilities of 92.80, 97.78, 94.82, and 91.43% in HepG2 cell lines. However, Pc, extract MeOH, extract H_2_O, GQDs MeOH, and CDs-H_2_O showed respective cell viabilities of 3.39, 92.80, 97.78, 94.82, and 91.43% in HepG2 cell lines. The percentage cell viability of different treatments compared to controls in Figure 8 shows that DMSO (0.1%) maintained viability at ~100.78%, confirming the non-toxic effect of the solvent. HCl (0.01%) reduced viability to ~92.35%, indicating minimal toxicity, while both CDs-H_2_O (91.43%) and GQDs-MeOH (94.81%) also showed reductions in cell viability that were less than 5%, suggesting the low toxicity of these nanomaterials. The extracts of H_2_O and MeOH showed respective cell viabilities of 97.78 and 92.79%, implying good biocompatibility, and are typically considered acceptable for non-toxic classification [11]. All tested samples retained high cell viability (>90%), indicating that they are largely biocompatible with only minor differences in cytotoxicity across treatments. These findings align with previous studies reporting low cytotoxicity of GQDs and plant extracts at moderate concentrations, supporting their potential biomedical applications [104,105].

#### 3.9.2. Viral Cytotoxicity: SARS-CoV-2 Papain-like Protease Assay

**Figure 9:**
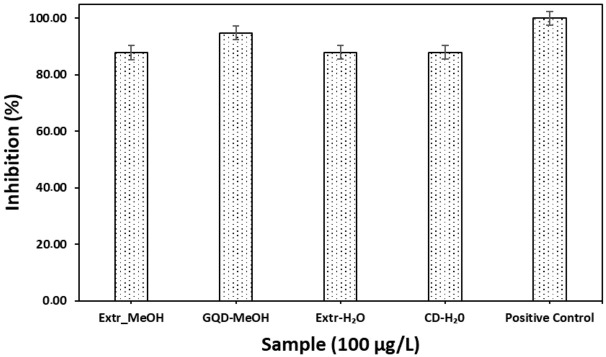
Percentage inhibition of *E. caffra*-synthesized nanoparticles and their extracts from H_2_O and MeOH.

The RFU-based fluorescence protease assays in Figure 9 show enzyme inhibition percentages of more than 80% for all the tested inhibitors when compared to the positive control RFU-based fluorescence protease assays, which showed 100% inhibition. Test samples, such as extract MeOH, extract H_2_O, and CDs-H_2_O, respectively, showed percentage inhibitions of 87.86 ± 4.75, 87.95 ± 0.77, and 87.95 ± 3.08% relative to GQDs MeOH (94.84 ± 0.94%).

The papain-like protease (PLPro) in SARS-CoV-2 is an enzyme whose specific enzymatic activity is detected by biosensors. Protease cleaves polyprotein precursors at specific sites, resulting in functional viral proteins [106]. The use of a substrate in biosensing occurs by cleavage of the protease at a specific peptide sequence within a substrate, resulting in a measurable signal, such as fluorescence or luminescence, to enable screening of viable antiviral inhibitors [18,107]. The known inhibitor, used as a positive control, demonstrated superior inhibitory effects when compared to the experimental inhibitor samples. The reduced fluorescence occurs when the protease binds to the inhibitor (Figure 9) and reduces its ability to cleave the substrate due to fewer fluorophores separating from the quencher. Plant extracts have been reported to prevent viral replication, inhibiting the RNA-dependent RNA polymerase (RdRp) protein complex (specifically the non-structural proteins (NSP)12, NSP7, and NSP8 subunits), which is a critical enzyme required for the replication and transcription of the viral RNA genome [108]. An in silico study on *Erythrina* species previously reported that flavonoid phytochemicals are potential SARS-CoV-2 3CL pro inhibitors [109].

In this study, the absence of flavonoids in the H_2_O extract of *E. caffra* when compared to the MeOH extract did not show a significant inhibition difference. However, GQDs showed twice as much improvement in inhibition relative to both extracts and CDs synthesized from the H_2_O extract. Some CDs have been reported to target viral enzymes like 3CLpro [109]. However, GQDs have been reported to inhibit viral entry and replication by interfering with the binding of the virus to host cell receptors [110,111]. The top part (membrane (M), envelope (E), and interior of the spike protein in the receptor-binding domain) maintains a positive charge over a broad range of pH [112,113]. The negative charge predominates on the surface exposed to the ACE2 receptor, patches of the viral envelope, and some areas on the S protein stalk [112,114]. In this study, CDs showed the highest zeta potential compared to GQDs. However, GQDs showed the most antiviral characteristics and lower toxicity compared to CDs. Thus, GQDs with high colloidal stability in complex biological environments, where interactions with proteins and ions can alter surface charge, can be used in biomedical applications [97]. This increased viral inhibition in GQDs compared to CDs is attributed to a combination of electrostatic surface interactions, physicochemical characteristics of the test inhibitor between the nanomaterials (or test sample) and the coronavirus, and the characteristics of the test inhibitor. This study has shown *E. caffra* extracts and respective nanomaterials (especially GQDs-MeOH) as potential SARS-CoV-2 antiviral agents.

## 4. Conclusions

This study successfully demonstrated the green synthesis of CDs and GQDs from *E. caffra* plant extraction in H_2_O and MeOH solvents. Both extracts showed the presence of multiple phytochemicals. However, flavonoid was absent in the H_2_O extract compared to MeOH. The synthesis of CDs and GQDs from respective extracts resulted in nanomaterials with enhanced or modified physicochemical and biological potential. Phytochemical profiling of *E. caffra* leaf extracts showed five classes of secondary metabolites. These phytochemical studies revealed that this plant can be a potential starting material for green synthesis of the carbon dots and treatment of SARS-CoV-2 papain-like protease (PLpro). Both the extracts and synthesized nanomaterials possess antioxidant characteristics. Spectroscopic and TEM characterization confirmed that the green synthesized nanoparticles are well-dispersed, colloidally stable, and dot-like, with diameters in the (~4–6 nm) range. The surface charges and zeta potential indicate that the synthesized nanoparticles were colloidally stable. The presence of functional groups and structural characteristics of nanomaterials confirmed GQDs to be highly crystalline compared to CDs and both extracts. Water-derived samples exhibited higher surface oxidation and exceptional colloidal stability, while methanol-derived samples displayed more hydrocarbon functionalities but relatively lower stability after synthesis. Both extracts and respective nanomaterials had minimal toxicities against HePG2. Importantly, GQDs demonstrated superior antiviral activity against the SARS-CoV-2 main protease, supporting their potential as inexpensive, plant-based nanomaterials for therapeutic development. Overall, these findings provide valuable evidence that *E. caffra* can serve as a sustainable precursor for functional nanomaterials with biomedical relevance.

## Figures and Tables

**Table 1:** Phytochemical screening, observation, and images of MeOH and H_2_O *E. caffra* plant extracts.

Phytochemical Test	Phytochemicals	Observation MeOH	Image	Observation H_2_O	Image
MeOH, H_2_O	Control	Lime	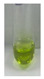	Pale brown	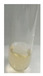
Wagner	Alkaloids	Reddish brown (+)	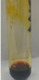	Reddish brown (+)	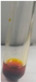
Shinoda	Flavonoids	Dark coloration (+)	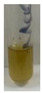	Pale brown (−)	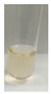
Tannins (FeCl_3_)	Tannins	Red brown (+)	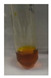	Brownish green (+)	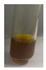
Salkowski	Steroids/Terpenoids	Brownish green (+)	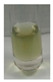	Reddish brown at the interface between two liquid layers (+)	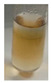
Phenols (FeCl_3_)	Phenols	Reddish Brown (+)	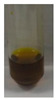	Reddish brown (+)	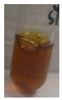

Negative (absent): −, and Positive (Present): +.

**Table 2:** Functional group, FTIR frequency, vibration, and phytochemical characteristics per sample.

Functional Group	FTIR Frequency (cm^−1^)	Reason	Phytochemical	Ref
(a) H_2_O extract				
C–O, C–O–C	1052.56	Stretching	C–O: Tannins and alkaloids C–O–C: Tannins	[24,54]
C–H, C–N, O–H	1392.25	C–H: Bending, O–H: in-plane bending, and C–N: stretch	steroids and terpenoids; O-H: phenolic and tannins	[65,67,68]
C=C	1594.94	Aromatic stretch	Tannins, phenols, and alkaloids	[67]
C=O	No peak			
C–H	2919.68, 2848.97	C–H stretch	Steroids and terpenoids	[68]
O–H	3261.50	Residual water, alcohol, or phenol stretch	Phenol and tannins	[65]
(b) CDs-H_2_O				
C–O, C–O–C	1052.56	Stretching	C–O: Tannins and alkaloids; C–O–C: Tannins	[24,54]
C=C	1526.44, 1631.54	Aromatic stretch	Tannins, phenols, and alkaloids	[67]
C=O	1737.87	Ester stretch	Flavonoids, phenolics, and glycosides	[65,67]
C–H	2919.68, 2850.65	C-H stretch	Steroids and terpenoids	[65,68]
O–H	3278.73	Alcohol or phenol stretch	Phenol and tannins	
(c) MeOH extract				
C–O, C–O–C	1052.56	Stretching	C–O: Tannins and alkaloids; C–O–C: Tannins	[24,54]
C–H, C–N	1367.86	C–H bending, C–N stretch	Aliphatic chains from terpenoids and amine groups from alkaloids	[65]
C=O	1737.87	Ester stretch	Flavonoids, phenolics, and glycosides	[65,67]
C–H	2919.68, 2849.81	C–H stretch	Steroids and terpenoids	[65,68]
O–H	3343.14	Alcohol or phenol stretch	Phenol and tannins	[55]
(d) GQDs-MeOH				
C–O, C–O–C	1052.56	Stretching	C–O: Tannins and alkaloids; C–O–C: Tannins	[24,54]
C–N, C–H	1355.66	C–N Stretch, C–H bending (scissoring or deformation)	C–N: Alkaloids; C–H: Terpenoids and steroids	[67,68]
C–C	1453.25	C–H bend		[55]
C=C	1607.14	Aromatic stretch	Tannins, phenols, and alkaloids	[67]
C=O	1737.87, 1703.25	Ester stretch, ketone stretch	Flavonoids, phenolics, and glycosides	[67,68]
C–H	2917.17, 2848.97	C–H stretch	Steroids and terpenoids	[68]
O–H	3318.74	Alcohol or phenol stretch	Phenol and tannins	[55,65]

**Table 3:** Characteristics of *E. caffra*’s H_2_O and MeOH extracts and green carbon nanomaterials.

Sample ID	Size (nm)			Zeta Potential (mV)	Absorbance (nm)	Emission (nm)	∅_*f*_	*d*_*spacing*_ (nm)	Scherrer (nm)	Percentage Crystallinity (%)	
	TEM	PDI	DLS							Crystalline	Amorphous
H_2_O Extract	_	_	_	−63.6	240	_	_	_	_	0	100
CDs-H_2_O	5.5 ± 1.5	0.2	437.5	+200.0	412	481	0.077	2.51	80.7	0	100
MeOH Extract	_	_	_	+49.8	270	_	_	_	_	5	95
GQDs-MeOH	4.0 ± 1.2	0.2	305.9	−12.6	411	484	0.190	0.08	80.9	100	0

Analysis was not done (_).

## Data Availability

The original contributions presented in this study are included in the article/Supplementary Material. Further inquiries can be directed to the corresponding authors.

## Supplementary Materials

The following supporting information can be downloaded at: https://www.mdpi.com/article/10.3390/ma19091841/s1.
