# Supplementary material for: Green Synthesis of Graphene Quantum Dots (GQDs) and Carbon Dots (CDs) Mediated with Erythrina caffra for Potential Antiviral Properties Against SARS-CoV-2
Source: Materials (Basel). 2026 Apr 30;19(9):1841. doi: 10.3390/ma19091841 (PMC13165359; doi:10.3390/ma19091841)
Supplement: Supplementary file 1 [file materials-19-01841-s001.zip › materials-4153827-supplementary.pdf]

materials

MDPI

Article

Green Synthesis of Graphene Quantum Dots (GQDs) and Carbon Dots (CDs) Mediated with *Erythrina caffra* for Potential Antiviral Properties Against SARS-CoV-2

Refilwe Matshitse [1,*], Boetie M. Buta [1,†], Nothando S. Mabasa [1,†], Bongeka S. Nkosi [1,†], Lebo A. Ramarope [1,†], Nhluvuko Vuma [1,†], Nomusa Sikhakhane [1], Tebogo Matlala [1], Charity E. Maepa [2], Sifiso A. Nsibande [3], Daniel Makanyane [4] and Xavier Siwe Noundou [1,*]

[1] Department of Pharmaceutical Sciences, School of Pharmacy, Sefako Makgatho Health Sciences University, Ga-Rankuwa, Pretoria 0204, South Africa; boetiemlungisi@gmail.com (B.M.B.); 201805593@swave.smu.ac.za (N.S.M.); nkosibongekasiphe@gmail.com (B.S.N.); leboramarope@gmail.com (L.A.R.); vumanhluvuko19@gmail.com (N.V.); nomusa.sikhakhane@smu.ac.za (N.S.); 201608154@swave.smu.ac.za (T.M.)

[2] Laboratory for Microscopy and Microanalysis, Faculty of Natural and Agricultural Sciences, University of Pretoria, Pretoria 0028, South Africa; charity.maepa@up.ac.za

[3] Chemistry Department, Faculty of Natural and Agricultural Sciences, University of Pretoria, Pretoria 0028, South Africa; sifiso.nsibande@up.ac.za

[4] Applied Radiation Department, South African Nuclear Energy Corporation Ltd., Brits 0240, South Africa; makanyanedaniella@gmail.com

* Correspondence: refilwemandla@yahoo.com (R.M.); xavier.siwenoundou@smu.ac.za (X.S.N.); Tel.: +27-12-521-5647 (X.S.N.)

† These authors contributed equally to this work.

Supplementary Data

S1 (a): Nonlinear curve fit processed with GaussAmp function selection for CDs-H_2O.

		Value	Standard Error
Intensity	y0	1,15759	0,14479
	xc	21,81534	0,00274
	w	0,20422	0,00522
	A	8,62195	0,149
	FWHM	0,48091	0,01228
	Area	4,41367	0,16066

Reduced Chi-sqr = 0,0149582334688
COD(R^2) = 0,99926533729329
Iterations Performed = 5
Total Iterations in Session = 5
Fit converged. Chi-Sqr tolerance value of 1E-9 was reached.
FWHM, Area are derived parameter(s).

Statistics

	Intensity
Number of Points	7
Degrees of Freedom	3
Reduced Chi-Sqr	0,01496
Residual Sum of Squares	0,04487
Adj. R-Square	0,99853
Fit Status	Succeeded(100)

Fit Status Code :
100 : Fit converged. Chi-Sqr tolerance value of 1E-9 was reached.

Summary

	y0		xc		w		A		FWHM	Area	Statistics	
	Value	Standard Error	Value	Standard Error	Value	Standard Error	Value	Standard Error	Value		Reduced Chi-Sqr	Adj. R-Square
Intensity	1,15759	0,14479	21,81534	0,00274	0,20422	0,00522	8,62195	0,149	0,48091	4,41367	0,01496	0,99853

ANOVA

		DF	Sum of Squares	Mean Square	F Value	Prob>F
Intensity	Regression	4	238,7879	59,69697	3990,91069	6,73046E-6
	Residual	3	0,04487	0,01496		
	Uncorrected Total	7	238,83277			
	Corrected Total	6	61,08204			

Fitted Curves Plot

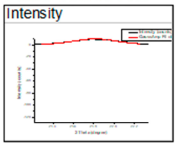

Residual vs. Independent Plot

S1 (b): Nonlinear curve fit processed with GaussAmp function selection for GQDs-MeOH.

		Value	Standard Error
Intensity	y0	0,4714	0,1319
	xc	21,83129	0,00331
	w	0,22844	0,00456
	A	13,21254	0,19121
	FWHM	0,53793	0,01074
	Area	7,56556	0,18484

Reduced Chi-sqr = 0,0458037020969
COD(R^2) = 0,99874540388343
Iterations Performed = 6
Total Iterations in Session = 6
Fit converged. Chi-Sqr tolerance value of 1E-9 was reached.
FWHM, Area are derived parameter(s).

Statistics

	Intensity
Number of Points	10
Degrees of Freedom	6
Reduced Chi-Sqr	0,0458
Residual Sum of Squares	0,27482
Adj. R-Square	0,99812
Fit Status	Succeeded(100)

Fit Status Code :
100 : Fit converged. Chi-Sqr tolerance value of 1E-9 was reached.

Summary

	y0		xc		w		A		FWHM	Area	Statistics	
	Value	Standard Error	Value	Standard Error	Value	Standard Error	Value	Standard Error	Value		Reduced Chi-Sqr	Adj. R-Square
Intensity	0,4714	0,1319	21,83129	0,00331	0,22844	0,00456	13,21254	0,19121	0,53793	7,56556	0,0458	0,99812

ANOVA

		DF	Sum of Squares	Mean Square	F Value	Prob>F
Intensity	Regression	4	483,69361	120,9234	2640,03554	9,48634E-10
	Residual	6	0,27482	0,0458		
	Uncorrected Total	10	483,96843			
	Corrected Total	9	219,05234			

Fitted Curves Plot

Intensity

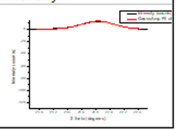

Residual vs. Independent Plot

S2 (a): Shapiro–Wilk one-way t-test statistical analysis of size distribution of (n >50), p >0.05 of CDs-H_2O.

(a)

One Sample t Test (3/17/2026 18:25:24)

Notes

X-Function	One Sample t Test
User Name	lebor
Time	3/17/2026 18:25:24
Data Filter	No

Input Data

	Data	Range
A	[Book1]Sheet1!A	[1*:58*]

Descriptive Statistics

	N	Mean	SD	SEM
A	58	5.30547	1.37544	0.1806

Test Statistics

	t Statistic	DF	Prob>|t|
A	-2.48208E-5	57	0.99998

Null Hypothesis: Mean = 5.30547
Alternative Hypothesis: Mean <> 5.30547
A: At the 0.05 level, the population mean is NOT significantly different from the test mean (5.30547).

Confidence Intervals for Mean

	Conf. Levels in %	Lower Limits	Upper Limits
A	95	4.94381	5.66712

S2 (b): Shapiro–Wilk one-way t-test statistical analysis of size distribution of (n >50), $p > 0.05$ for GQDs-MeOH.

(b)

One Sample t Test (3/17/2026 18:28:33)

Notes

X-Function	One Sample t Test
User Name	lebor
Time	3/17/2026 18:28:33
Data Filter	No

Input Data

	Data	Range
A	[Book1]Sheet1!A	[1*:61*]

Descriptive Statistics

	N	Mean	SD	SEM
A	61	3.98385	1.21918	0.1561

Test Statistics

	t Statistic	DF	Prob>|t|
A	1.57529E-5	60	0.99999

Null Hypothesis: Mean = 3.98385
Alternative Hypothesis: Mean <> 3.98385
A: At the 0.05 level, the population mean is NOT significantly different from the test mean (3.98385).

Confidence Intervals for Mean

	Conf. Levels in %	Lower Limits	Upper Limits
A	95	3.67161	4.2961
